# Siderophores: More than Stealing Iron

**DOI:** 10.1128/mBio.01906-16

**Published:** 2016-11-15

**Authors:** Judith Behnsen, Manuela Raffatellu

**Affiliations:** a Department of Microbiology and Molecular Genetics, University of California Irvine School of Medicine, Irvine, California, USA; b Institute for Immunology, University of California Irvine School of Medicine, Irvine, California, USA

## Abstract

Siderophores are small molecular iron chelators that are produced by microbes and whose most notable function is to sequester iron from the host and provide this essential metal nutrient to microbes. Recent studies have proposed additional, noncanonical roles for siderophores, including the acquisition of noniron metals and modulation of host functions. Recently, Holden et al. (V. I. Holden, P. Breen, S. Houle, C. M. Dozois, and M. A. Bachman, mBio 7:e01397-16, 2016, http://dx.doi.org/10.1128/mBio.01397-16) showed that siderophores secreted by *Klebsiella pneumoniae* during lung infection induce stabilization of the transcription factor HIF-1α, increase the expression of proinflammatory cytokines in the lung, and promote dissemination of *K. pneumoniae* to the spleen. Thus, their study demonstrated novel roles for siderophores *in vivo*, beyond iron sequestration. The interaction of siderophores with host cells further promotes the pathogenicity of *K. pneumoniae* and is likely relevant for other pathogens that also secrete siderophores in the host.

## COMMENTARY

Iron is an essential nutrient for the host as well as for most microbes. In the host, free iron levels are extremely low (e.g., <10^−24^ M for Fe^3+^ in serum), as the metal is largely bound to proteins, and iron is further limited during infection through a process known as nutritional immunity (1). To overcome nutritional immunity, some bacteria and fungi produce siderophores, which are small molecules that chelate iron (2). Enterobactin (also known as enterochelin) is a catecholate siderophore produced by both commensal and pathogenic *Enterobacteriaceae* and has greater affinity for iron than host molecules, such as transferrin and lactoferrin (3). To combat this, the host has evolved to produce lipocalin-2 (also known as neutrophil gelatinase-associated lipocalin, siderocalin, and 24p3), an antimicrobial protein that binds to iron-laden enterobactin, thereby preventing its reuptake by bacteria (4). Nevertheless, in the “tug of war” for iron, many enteric pathogens have acquired additional mechanisms to evade lipocalin-2 activity, in particular by producing and acquiring “stealth siderophores” that are not bound by lipocalin-2 (5). Examples of stealth siderophores include salmochelin (a C-glucosylated derivative of enterobactin) and yersiniabactin (a mixed-type siderophore). These molecules allow pathogens to evade lipocalin-2-mediated iron starvation and thereby confer an advantage to pathogens during infection and inflammation, when lipocalin-2 is highly expressed (6, 7).

The repertoire of siderophores varies among different microbial species and even among different strains. Indeed, various combinations of siderophores (e.g., enterobactin, salmochelin, yersiniabactin) are found among clinical isolates of *Klebsiella pneumoniae* (8), a member of the *Enterobacteriaceae* that causes pneumonia, urinary tract infection, and septicemia, largely in hospitalized patients. This diversity in siderophores impacts the replicative niche of *K. pneumoniae* in the host (8, 9) and suggests that siderophores contribute to pathogenesis via different mechanisms (10).

Long known to scavenge iron during infection, recent studies have highlighted additional siderophore functions. For example, enterobactin, but not yersiniabactin, appears to protect bacteria from oxidative stress (11). On the other hand, yersiniabactin has been shown to act as a chelator of additional metals, including copper and zinc. Strains such as uropathogenic *Escherichia coli* appear to use yersiniabactin’s copper-binding properties as a mechanism to resist copper toxicity (12), whereas yersiniabactin’s zinc-binding ability allows *Yersinia pestis* to resist zinc limitation in a septicemic plague mouse model (13).

In addition to promoting microbial growth by binding metals, there is emerging evidence that siderophores can modulate the host response. HIF-1α is a transcription factor that plays pivotal roles during infection (14). It was previously shown that siderophores secreted by enteric pathogens cause hypoxia-dependent activation of HIF-1α in the Peyer’s patches and in human epithelial and endothelial cells (15). Previously, Holden et al. demonstrated that enterobactin stabilizes HIF-1α in respiratory cells *in vitro*, thereby inducing expression of proinflammatory cytokines and enhancing lipocalin-2-mediated inflammation (16). In a more recently published *mBio* article by Holden et al. (17), the authors hypothesized that siderophores secreted by *K. pneumoniae* during lung infection can also have proinflammatory effects by interacting with host cells, thereby promoting pathogenicity during pneumonia.

To determine whether *K. pneumoniae* siderophores also have proinflammatory effects *in vivo*, Holden and colleagues employed a mutant that can secrete, but not take up, siderophores (a *tonB* mutant). When the *K. pneumoniae* *tonB* mutant was administered to mice, mass spectrometry of lung homogenates confirmed the presence of salmochelin and yersiniabactin. Moreover, these authors showed that the *tonB* mutant induced expression of wild-type proinflammatory cytokine levels at early time points postinfection. In comparison to an isogenic strain unable to secrete siderophores (an *entB ybtS tonB* mutant), the *tonB* mutant exhibited greater dissemination from the lung to the spleen. Interestingly, siderophores appeared to only induce a subset of proinflammatory proteins, including interleukin-6 and the neutrophil chemoattractants CXCL1 and CXCL2, whereas induction of other proinflammatory molecules (interleukin-1β [IL-1β], macrophage inhibitory protein-3α) was independent of siderophore secretion. Analysis of strains with mutations in different combinations of siderophores indicated that all siderophores contributed to the induction of host inflammation and promoted the dissemination of *K. pneumoniae* to the spleen. Both phenotypes were independent of lipocalin-2 expression, as similar levels of pathogen dissemination and host expression of proinflammatory cytokines were observed in both wild-type and lipocalin-2-deficient mice. This is in contrast to prior *in vitro* observations, where lipocalin-2 enhanced production of cytokines in airway epithelial cells (16), a difference that Holden et al. suggested might be the result of redundant signals compensating for lipocalin-2 deficiency *in vivo*.

As their prior study indicated that siderophores stabilize HIF-1α in respiratory cells (16), Holden and colleagues sought to investigate whether this occurs *in vivo*. To this end, they employed a transgenic mouse model that expresses a luciferase fusion to the oxygen-dependent domain (ODD) of HIF-1α, which becomes stabilized under low-oxygen or low-iron conditions. By performing these elegant experiments, Holden et al. demonstrated that siderophores secreted in the lung by *K. pneumoniae* lead to greater HIF-1α stabilization and that greater HIF-1α stabilization correlates with increased *K. pneumoniae* dissemination to the spleen. These authors then carried out a key experiment to show a functional link between siderophore-mediated HIF-1α stabilization and bacterial dissemination: infection of transgenic mice in which *Hif1a* deletion was induced postnatally only in lung epithelial cells. In *Hif1a-*deficient mice, *K. pneumoniae* dissemination to the spleen was significantly reduced, demonstrating a role for siderophore-dependent HIF-1α stabilization in promoting dissemination of *K. pneumoniae*.

**FIG 1:**
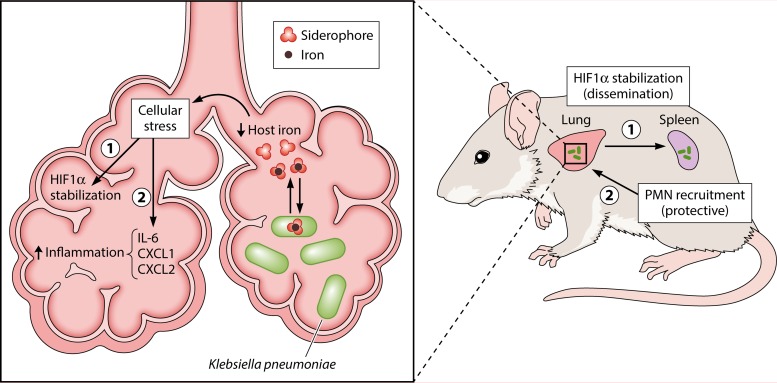
Working model. When *Klebsiella pneumoniae* enters the lung, the host responds to the infection by restricting iron availability. In order to obtain the iron necessary for its growth, *K. pneumoniae* secretes siderophores, which can bind iron with greater affinity than host proteins. The resulting lower levels of host iron induce stress in host cells. This stress response has two consequences for the progression of the infection: (i) HIF-1α stabilization, which ultimately leads to bacterial dissemination to peripheral organs like the spleen; (ii) the secretion of proinflammatory cytokines IL-6, CXCL1, and CXCL2. The chemokines CXCL1 and CXCL2 recruit neutrophils (polymorphonuclear lymphocytes [PMN]) to the site of infection, a response that is crucial for host protection from *K. pneumoniae* infection.

The primary significance of their study is its demonstration of a noncanonical role for siderophores *in vivo* during *K. pneumoniae* infection. In addition to providing iron to the pathogen, siderophores also promote *K. pneumoniae* dissemination to the spleen by inducing stabilization of HIF-1α in lung epithelial cells (Fig. 1). The study also demonstrated that HIF-1α enhances susceptibility to *K. pneumoniae* infection. Future investigations will need to address the mechanism by which epithelial HIF-1α promotes *K. pneumoniae* dissemination. An additional response induced by siderophores is the expression of proinflammatory cytokines, such as IL-6 and CXC chemokines, which was independent of HIF-1α in lung epithelial cells. These chemokines promote the recruitment of neutrophils, which in turn confer protection during *K. pneumoniae* infection but also contribute to pathology. Thus, therapeutic interventions aiming to inactivate siderophores may be beneficial to the host not only by inhibiting pathogens from acquiring iron but also by preventing pathogen dissemination while limiting pathology through modulating the immune system.

## References

[1] Cassat JE, Skaar EP 2013 Iron in infection and immunity. Cell Host Microbe 13:509–519. doi:10.1016/j.chom.2013.04.010. 23684303 PMC3676888

[2] Crosa JH, Walsh CT 2002 Genetics and assembly line enzymology of siderophore biosynthesis in bacteria. Microbiol Mol Biol Rev 66:223–249. doi:10.1128/MMBR.66.2.223-249.2002. 12040125 PMC120789

[3] Raymond KN, Dertz EA, Kim SS 2003 Enterobactin: an archetype for microbial iron transport. Proc Natl Acad Sci U S A 100:3584–3588. doi:10.1073/pnas.0630018100. 12655062 PMC152965

[4] Flo TH, Smith KD, Sato S, Rodriguez DJ, Holmes MA, Strong RK, Akira S, Aderem A 2004 Lipocalin 2 mediates an innate immune response to bacterial infection by sequestrating iron. Nature 432:917–921. doi:10.1038/nature03104. 15531878

[5] Fischbach MA, Lin H, Liu DR, Walsh CT 2006 How pathogenic bacteria evade mammalian sabotage in the battle for iron. Nat Chem Biol 2:132–138. doi:10.1038/nchembio771. 16485005

[6] Bachman MA, Miller VL, Weiser JN 2009 Mucosal lipocalin 2 has pro-inflammatory and iron-sequestering effects in response to bacterial enterobactin. PLoS Pathog 5:e1000622. doi:10.1371/journal.ppat.1000622. 19834550 PMC2757716

[7] Raffatellu M, George MD, Akiyama Y, Hornsby MJ, Nuccio SP, Paixao TA, Butler BP, Chu H, Santos RL, Berger T, Mak TW, Tsolis RM, Bevins CL, Solnick JV, Dandekar S, Bäumler AJ 2009 Lipocalin-2 resistance confers an advantage to *Salmonella enterica* serotype Typhimurium for growth and survival in the inflamed intestine. Cell Host Microbe 5:476–486. doi:10.1016/j.chom.2009.03.011. 19454351 PMC2768556

[8] Bachman MA, Oyler JE, Burns SH, Caza M, Lépine F, Dozois CM, Weiser JN 2011 *Klebsiella pneumoniae* yersiniabactin promotes respiratory tract infection through evasion of lipocalin 2. Infect Immun 79:3309–3316. doi:10.1128/IAI.05114-11. 21576334 PMC3147564

[9] Bachman MA, Lenio S, Schmidt L, Oyler JE, Weiser JN 2012 Interaction of lipocalin 2, transferrin, and siderophores determines the replicative niche of *Klebsiella pneumoniae* during pneumonia. mBio 3:e00224-11. doi:10.1128/mBio.00224-11. 23169997 PMC3509427

[10] Holden VI, Bachman MA 2015 Diverging roles of bacterial siderophores during infection. Metallomics 7:986–995. doi:10.1039/c4mt00333k. 25745886

[11] Achard ME, Chen KW, Sweet MJ, Watts RE, Schroder K, Schembri MA, McEwan AG 2013 An antioxidant role for catecholate siderophores in *Salmonella*. Biochem J 454:543–549. doi:10.1042/BJ20121771. 23805839

[12] Chaturvedi KS, Hung CS, Crowley JR, Stapleton AE, Henderson JP 2012 The siderophore yersiniabactin binds copper to protect pathogens during infection. Nat Chem Biol 8:731–736. doi:10.1038/nchembio.1020. 22772152 PMC3600419

[13] Bobrov AG, Kirillina O, Fetherston JD, Miller MC, Burlison JA, Perry RD 2014 The *Yersinia pestis* siderophore, yersiniabactin, and the ZnuABC system both contribute to zinc acquisition and the development of lethal septicaemic plague in mice. Mol Microbiol 93:759–775. doi:10.1111/mmi.12693. 24979062 PMC4132657

[14] Nizet V, Johnson RS 2009 Interdependence of hypoxic and innate immune responses. Nat Rev Immunol 9:609–617. doi:10.1038/nri2607. 19704417 PMC4343208

[15] Hartmann H, Eltzschig HK, Wurz H, Hantke K, Rakin A, Yazdi AS, Matteoli G, Bohn E, Autenrieth IB, Karhausen J, Neumann D, Colgan SP, Kempf VA 2008 Hypoxia-independent activation of HIF-1 by enterobacteriaceae and their siderophores. Gastroenterology 134:756–767. doi:10.1053/j.gastro.2007.12.008. 18325389

[16] Holden VI, Lenio S, Kuick R, Ramakrishnan SK, Shah YM, Bachman MA 2014 Bacterial siderophores that evade or overwhelm lipocalin 2 induce hypoxia inducible factor 1α and proinflammatory cytokine secretion in cultured respiratory epithelial cells. Infect Immun 82:3826–3836. doi:10.1128/IAI.01849-14. 24980968 PMC4187820

[17] Holden VI, Breen P, Houle S, Dozois CM, Bachman MA 2016 *Klebsiella pneumoniae* siderophores induce inflammation, bacterial dissemination, and HIF-1α stabilization during pneumonia. mBio 7(5):e01397-16. doi:10.1128/mBio.01397-16. 27624128 PMC5021805

